# Dynamic sourcing strategies for supply disruptions under consumer stockpiling

**DOI:** 10.1007/s40747-021-00520-9

**Published:** 2021-09-15

**Authors:** Shanshan Li, Yong He, Li Zhou

**Affiliations:** 1 School of Finance, Nanjing Audit University, Nanjing, 211815 China; 2 School of Economics and Management, Southeast University, Nanjing, 210096 China

**Keywords:** Supply disruption management, Dynamic sourcing strategies, Customer stockpiling behavior

## Abstract

This paper considers a make-to-order system where production gets disrupted due to a random supply failure. To avoid potential stock-out risk and responding price increase during disruption, customers might decide to stockpile extra units for future consumption. We investigate the contingent sourcing strategy for the manufacturer to cope with the disruption. To this end, we first discuss the optimal post-disruption stockpiling decision for customers. In view of expected disruption duration, price rise, and inventory holding cost, three types of stockpiling behavior are analytically provided for the customers: non-stockpiling, gradual stockpiling, and instantaneous stockpiling. Next, a model is formulated to optimize the joint decision of contingent sourcing time and quantity, with the objective of maximizing profit expectation. Finally, by conducting numerical analysis, we generate further insights into the role of relative factors and provide specific managerial suggestions on how to adapt dynamic contingent sourcing strategies to alleviate different disruptions, under different market environments and customer behaviors.

## Introduction

Unexpected events (such as natural disasters) appearing at any nodes could lead to partial or full breakdowns of supply chain systems, resulting in devastating short-term and long-term losses [1]. For example, during the early phase of the COVID-19 pandemic, 94% of the Fortune 1000 companies have been undergone coronavirus-driven SC disruptions by March 2020 [2].

In designing an effective supply risk mitigation strategy, it is critical to understand and precisely capture the post-disruption demand. However, to the best of our knowledge, the existing literature did not pay enough attention to this direction. In the context of supply chain disruption management, customers’ reaction is commonly captured as backorders or lost sales, and identified as deterministic demands or indeterministic demands with given distributions [3]. In fact, after the occurrence of a disruption, consumers often stockpile products through alternative channels to avoid future shortages or price rises, especially during the disruptions caused by natural disasters. For instance, in 2011, after the radioactive leak at Japan’s Fukushima nuclear plant, consumers went on panic buying various groceries to prevent radiation poisoning. More recently, during the COVID-19 pandemic, stockpiling essential groceries such as food and toilet paper, have been emerging around the world [4].

The problem regarding consumer stockpiling behavior has been gaining growing attention in both marketing and supply chain research. In specific to the context of supply chain disruption management, the existing literature starts to identify changes in customers’ purchasing behavior, triggered by the fear of future supply shortages or price rises. However, to date, the research is still in its infancy in establishing mitigation tactics for manufacturers/retailers, considering customer stockpiling decisions in the presence of supply disruptions. We thus attempt to fill this gap in this study.

To this end, we consider an MTO production system where a manufacturer sources from a single supplier and competitive manufacturers provide alternative products in the market. After the occurrence of a supply disruption, production pauses immediately if no countermeasure is taken. As a reaction, customers switch to the competitive manufacturers and might stockpile by purchasing more alternative products than their real-time needs. Driven by the shortage of products, the selling price of alternative products provided by competitors increases over time. To hedge against the negative impacts, the manufacturer employs a supply-side contingent sourcing. Sufficient surveys have pointed out that contingent sourcing is a widely utilized emergency countermeasure in practical [5]. Several essential research questions arise accordingly: driven by the fear that price will continue growing during the shortage, how do the consumers dynamically adjust their purchasing behavior? What is the optimal joint decision of contingent sourcing time and quantity, taking into account the dynamic process of customers’ stockpiling?

In answering these two questions, our present study contributes to the existing literature with two unique folds. First, in view of disruption durations and price rises, we provide three patterns of optimal post-disruption stockpiling decisions for the customers: non-stockpiling, gradual stockpiling, and instantaneous stockpiling. The corresponding conditions under which they should choose the three decisions are also presented. Second, we propose dynamic contingent sourcing strategies that identify optimal joint decision of time and quantity for the manufacturer. The strategies enable him/her to arrive at more profits during random supply disruptions. We also generate specific managerial insights on how to adjust the strategies with respect to relative factors including inventory holding cost, mean value of disruption duration, and prices before and after the occurrence of disruption.

The remainder of the paper is organized as follows. In “Related literature”, the related work is briefly reviewed. “Problem description” describes the problem. Customer stockpiling behavior is analyzed in “Customers’ stockpiling behavior during disruption”. Dynamic contingent sourcing strategies are developed in “Manufacturer’s contingent sourcing policy”. Finally, “Conclusions” gives conclusions and future research directions.

## Related literature

To alleviate the negative impact caused by supply chain failures, a fruitful of tactics have been proposed, for instance, supplier diversification, contingent sourcing, inventory buffering, production scheduling and recovery, customer compensation, etc. [6–10]. The relevant literature mainly falls into two streams: contingent sourcing policy, and post-disruption demand identification considering stockpiling behavior.

### Contingent sourcing policy

Contingent sourcing is a default countermeasure for disruption mitigation and attracts extensive work in academics. Under contingent sourcing, the manufacturer places an emergency purchase from secondary/backup suppliers in the event of a failure at its main supplier [11]. Accordingly, related research mainly focuses on optimizing the order placement in emergency purchases. For example, considering two competing manufacturers, [12] and [13] investigate optimal order allocation decisions under emergency procurement strategy. Subsequent studies extend this optimization problem in various directions, such as incorporating price competition [14], focusing on the supply chains of specific industries, etc. [15, 16].

The direction closer to our study is the extension to dynamic aspects. For instance, by modeling a time-dependent supply failure through a standby approach, [17] proposes an optimal sourcing strategy considering a combination of supply risk, a ratio of operational cost vs. loss, and supply period length. Modeling dynamic disruption risks as chains, [18] develops the optimal backup flexibility design for resilience increase. Some work concerns the optimal sourcing time on top of the optimal sourcing quantity decision. Taking an uncertain lead time into consideration, [19] investigates the optimal timing and quantity of an emergency order under a periodic review inventory system. By forecasting customers’ reactions through a demand-learning model, [20] identifies the optimal contingent sourcing time to minimize disruption costs.

As can be seen from the brief review above, little research has been done on dynamic contingent sourcing strategies, taking into account consumer stockpiling behavior. In fact, a few studies point out that consumer stockpiling behavior might exacerbate the impact caused by supply disruption [21]. Thus, we intend to bridge this research gap. The literature most relevant to our paper is the ones by [22] and [23]. They examine optimal sourcing strategy for the retailer who sells a product over two periods, considering that customers might hoard one extra unit of the product in period 1 to prevent the potential shortage in period 2. In the present paper, we focus on the optimal joint decision of contingent sourcing time and quantity, considering a supply disruption that will last a random length.

### Post-disruption demand identification considering stockpiling

Post-disruption demand is commonly identified as various types of stochastic demand distributions or deterministic demand functions [24, 25]. Nonetheless, another stream of studies questions that such methodologies might be ineffective to capture the fast-changing characteristics of customers’ reactions, thus develops forecasting methods to further identify post-disruption demand [26]. For example, [27] establishes an improved model of grey neural networks for demand prediction, in the context of transportation disruption.

In specific to customer stockpiling (or panic buying) behavior that is frequently observed after the appearance of unexpected events such as natural disasters, the focus of related research is commonly confined to investigating changes in customers’ purchasing behavior, driven by the supply failures of goods and services [28–31]. For example, considering both limited quantity scarcity and limited time scarcity, [32] identifies consumers’ panic buying behaviors during COVID-19 through the models of Stimuli-Organism-Response and Competitive Arousal. Using archival retail scanner data and real‐time data, [33] studies consumer precautionary stockpiling behavior before landfalls of the hurricane and points out three types of contributing factors including the characteristics of supply-side, demand-side, and disaster itself. On the other hand, the present topic also attracts great attention in marketing literature, concentrating on two questions: how forward-looking consumers stockpile non-perishable products under changing sales prices [34], and how such behavior affects the pricing decision of suppliers [35]. Guo and Villas-Boas [36] provides a systematic overview of the existing research on panic buying.

However, to date, little literature has been found exploring the optimal stockpiling behavior for customers in the presence of supply failures. One of our objectives is to address this research gap.

## Problem description

Notations are defined in Table 1.

**Table 1:** Notations

	Notations	Description
Decisions	$Q$	Contingent sourcing quantity
	${t}^{*}$	Contingent sourcing time
Parameters	$T$	Disruption duration
	$E(T)$	Mean value of the random variable $T$
	${T}_{\mathrm{P}}$	Time when customers stop stockpiling
	$p(t)$	Selling price per unit of product (including alternative product)
	$v$	Consumers' valuation of one unit of product
	${v}_{0}$	Consumers' valuation of one unit of an alternative product, ${v}_{0}<v$
	$d\left(t\right)$	Demand rate at time $t$ after the occurrence of a disruption
	${c}_{\mathrm{s}}$	Unit extra sourcing cost from the emergency supplier
	${c}_{\mathrm{h}}$	Unit inventory holding cost per unit of time for customers
	${c}_{\mathrm{H}}$	Unit inventory holding cost per unit of time for the manufacturer
	${I}_{\mathrm{C}}\left(t\right)$, $I\left(t\right)$	Inventory held by customers and the manufacturer

A firm produces and sells a single product to customers, and sources from a regular supplier who is unreliable. There is an emergency supplier who is reliable and expensive, and a competitive manufacturer selling alternative products. The demand rate of finished products is deterministic and normalized to be “1”. The firm practices make-to-order manufacturing, i.e., no inventory of finished products is hoarding in the system. Before the appearance of supply disruption, the production is realized at the demand rate “$1$”. Without loss of generality, we assume that a supply disruption occurs at time ‘0’ and will last $T$ periods. Here $T$ is a random variable with a mean value $E(T)$. Due to the event of supply disruption, the selling price of the product increases over time, denoted as:

1 $$p\left(t\right)={p}_{0}+\theta t.$$

where $0\le t\le T$. ${p}_{0}$ represents the selling price before the supply disruption occurs, and $\theta$ stands for the rate of the price increase.

Without the adoption of countermeasures, the production stops immediately leading to a stock-out for customers. Facing stock-out, customers purchase alternative products from the competitive manufacturer. To avoid a loss of utility caused by the later increase in price, customers may stockpile by purchasing more than needed during the early stages.

In this study, we examine two questions: how the relative factors such as expected disruption length and price rise affect the consumers’ post-disruption purchasing behavior, and what is the optimal sourcing time and quantity for the manufacturer to cope with the supply disruption, taking customers’ stockpiling dynamics into consideration.

Next, we start by exploring the customers’ purchasing behavior during disruption.

## Customers’ stockpiling behavior during disruption

Based on the expectation $E(T)$ of the supply disruption duration, customers decide to purchase $d\left(t\right)$ units of the alternative products at time $t$, arriving at an expected utility $U$:

2 $$U={\int }_{0}^{E(T)}[{v}_{0}-p(t)]d\left(t\right)\mathrm{d}t-{c}_{\mathrm{h}}{\int }_{0}^{E\left(T\right)}{I}_{\mathrm{C}}\left(t\right)\mathrm{d}t.$$

The first term of (2) captures the expected utility that customers perceive from the alternative products during disruption, and the second term presents the utility loss caused by holding inventory. Where the inventory dynamics ${I}_{\mathrm{C}}(t)$ is formulated as:

3 $$ I_{{\text{C}}} \left( t \right) = \left\{ {\begin{array}{*{20}l} {\mathop \smallint \limits_{0}^{t} \left[ {d\left( \tau \right) - 1} \right]{\text{d}}\tau , } \hfill & {{\text{if}}\; t \in \left[ {0,T_{{\text{P}}} } \right] \cup \left[ {T_{{\text{P}}} ,T_{0} } \right]; } \hfill \\ {\mathop \smallint \limits_{0}^{{T_{0} }} \left[ {d\left( \tau \right) - 1} \right]{\text{d}}\tau - \left( {t - T_{{\text{P}}} } \right),} \hfill & {{\text{if}}\; t \in \left[ {T_{0} ,E\left( T \right)} \right].} \hfill \\ \end{array} } \right. $$

As indicated in (3), the inventory dynamics could fall into three phases, dependent on the pattern under which customers conduct their purchasing behavior. In the first phase $[0,{T}_{\mathrm{P}}]$, the inventory keeps increasing due to $d\left(t\right)>1$, that is, customers are stocking up. Then, in the second phase $[{T}_{\mathrm{P}}, {T}_{0}]$, in view of the inventory accumulated during the previous phase, customers might choose to purchase less than their needs, i.e., $0<d\left(t\right)\le 1$. Thus, inventory is required to be gradually consumed at the rate of $1-d\left(t\right)$. Lastly, in the third phase $[{T}_{0},E(T)]$, no purchase is conducted. As a result, customers’ inventory is consumed at the maximum rate of “1”.

Given (2), the customers’ optimal stockpiling decision can be formulated as the following problem.

4 $$ \max \;U. $$

5 $$ {\text{Subject to}}\mathop \smallint \limits_{0}^{E\left( T \right)} d\left( t \right){\text{d}}t = E\left( T \right). $$

6 $$d\left(t\right)\ge 0.$$

7 $$d\left(0\right)>1.$$

The objective function (4) maximizes the customers’ expected utility. Equation (5) guarantees that the inventory held by customers is entirely depleted at the end of the disruption, i.e., ${I}_{\mathrm{C}}\left(t\right)=0$ if $t=E(T)$. Equation (6) ensures the non-negativity of customers’ purchase quantity. Equation (7) illustrates that the customers’ stockpiling behavior occurs during the early phases of supply disruption.

To generate further insights, we assume that the customers’ stockpiling behavior follows a linear pattern, i.e., $d\left(t\right)=b-at$. Here, $a, b\ge 0$. Note that, by doing so, we extend the assumption utilized in most related literature that customers’ stockpiling behavior is confined to a one-time purchase [22, 23].

**Fig. 1:**
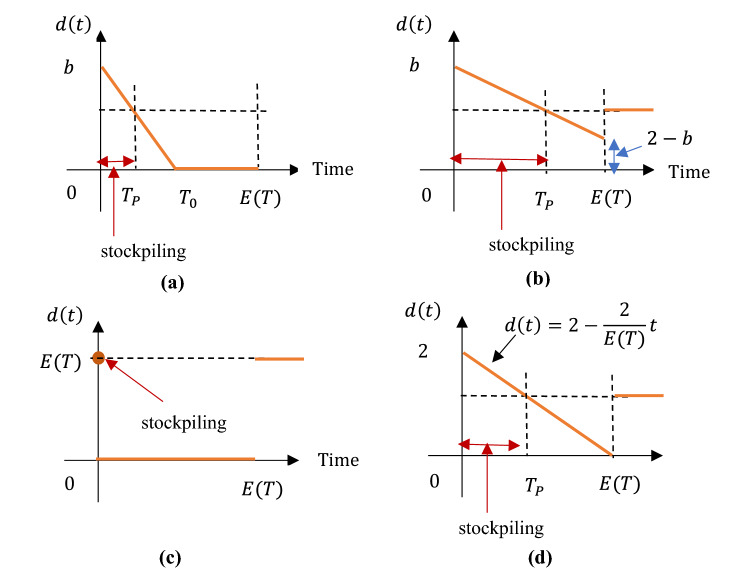
Customers’ possible stockpiling behaviors under the linear assumption

As depicted in (Fig. 1a, b), customers’ behavior might fall into two patterns under the present linear assumption.

In **Pattern 1** where ${T}_{0}\le E(T)$, customers stockpile at a large speed during the first phase $[0, {T}_{P}]$ so that there is no need for them to purchase any products at the late period $[{T}_{0},E(T )]$.

In **Pattern 2** where ${T}_{0}>E(T)$, the stocking speed is relatively small, thus the accumulated inventory does not suffice to fulfill the future consumption that appears after the time point ${T}_{\mathrm{P}}$. Therefore, the purchase is required during the entire interruption, i.e., $d(t)>0$ for all $t\in [0,E(T )]$.

In particular, in view of the customers’ stocking speed, these two patterns could reach three boundaries, referred to as **Cases 1–3** in the following.

(i) In **Case 1** where $d\left(t\right)=1$: no stockpiling behavior appears.
(ii) In **Case 2** where $d\left(t\right)={d}_{1}(t)=2-\frac{2}{E(T)}t$: customers stockpile at a medium speed. To be specific, to fulfill their real-time consumption rate “1”, they choose to purchase “2” units of the products at the beginning of the supply failure, then gradually reduce the purchase rate until $d\left(t\right)$ reaches zero at the expected end time $E(T)$ of disruption (Fig. 1d).
(iii) In **Case 3** where $d\left(0\right)=b=E(T)$: customers stockpile the total expected inventory required for the entire disruption immediately at the initial time $t=0$. In other words, to avoid the mark-up, customers purchase zero product thereafter, i.e., $d\left(t\right)=0$ for $t>0$ (Fig. 1c).

Solving problem (4)–(7) under the linear assumption, we find that the customers’ optimal stockpiling decisions are achieved at the above three boundaries, as shown in Table 2.

**Table 2:** Customers’ optimal stockpiling behavior

Cases	Conditions		Customers’ optimal stockpiling behavior
1	$\theta <{c}_{\mathrm{h}}$	$E(T)<\frac{3}{4}+\frac{{c}_{\mathrm{h}}}{4\theta }$	Non-stockpiling, $d\left(t\right)=1$
2		$E(T)>\frac{3}{4}+\frac{{c}_{\mathrm{h}}}{4\theta }$	Gradual stockpiling, $d\left(t\right)={d}_{1}(t)=2-\frac{2}{E(T)}t$
	$\theta >{c}_{\mathrm{h}}$	$E(T)>\frac{3}{2}-\frac{{c}_{\mathrm{h}}}{2\theta }$	
3		$E(T)<\frac{3}{2}-\frac{{c}_{\mathrm{h}}}{2\theta }$	Instantaneous stockpiling, $d\left(0\right)=E(T)$

The calculation of Table 2 is detailed in the appendix.

As indicated in Table 2, the customers’ optimal stockpiling behavior is critically linked to the following factors: the rate $\theta$ of price increase, the inventory holding cost ${c}_{\mathrm{h}}$, and the disruption length represented by the mean value $E(T)$. The results provide the following suggestions for customers. First, customers only refrain from stockpiling (i.e., Case 1) in the event that there will be no substantial price increase and the disruption lasts short. Second, if the disruption lasts significantly short and the selling price will rise sharply, it is superior for the customers to instantly stockpile a large number of products at the beginning (i.e., Case 3). Except for these two special circumstances, the optimal purchasing decision falls into gradual stockpiling (i.e., Case 2).

## Manufacturer’s contingent sourcing policy

In this section, based on customers’ stockpiling behavior, we propose dynamic contingent sourcing policies for the manufacturer, identifying the optimal sourcing quantity and time. Note, as indicated in Table 2, the likelihood of Case 3 is rare, we then focus on Cases 1–2 in the following.

### Manufacturer’s profit

Suppose the manufacturer reroutes to a secondary source at the time ${t}^{*}$ and purchases $Q$ units of raw material to alleviate the disruption and customers’ stockpiling. With contingent sourcing, the manufacturer resumes production immediately. As a result, the remaining demand $d\left(t\right)$ can be partially or fully satisfied, depending on the quantity $Q$. Note that, compared with the alternative products provided by competitors in the market, customers evaluate a larger utility $v$ on the product provided by the manufacturer. Thus, we assume that the customers will choose to order from the manufacturer in priority.

**Fig. 2:**
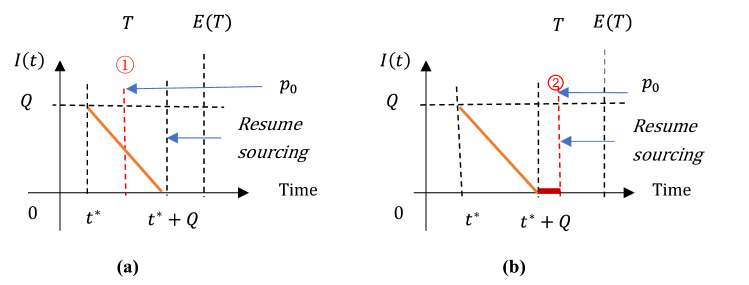
Manufacturer’s inventory $I\left(t\right)$ under contingent sourcing $Q({t}^{*})$ in Case 1

**Fig. 3:**
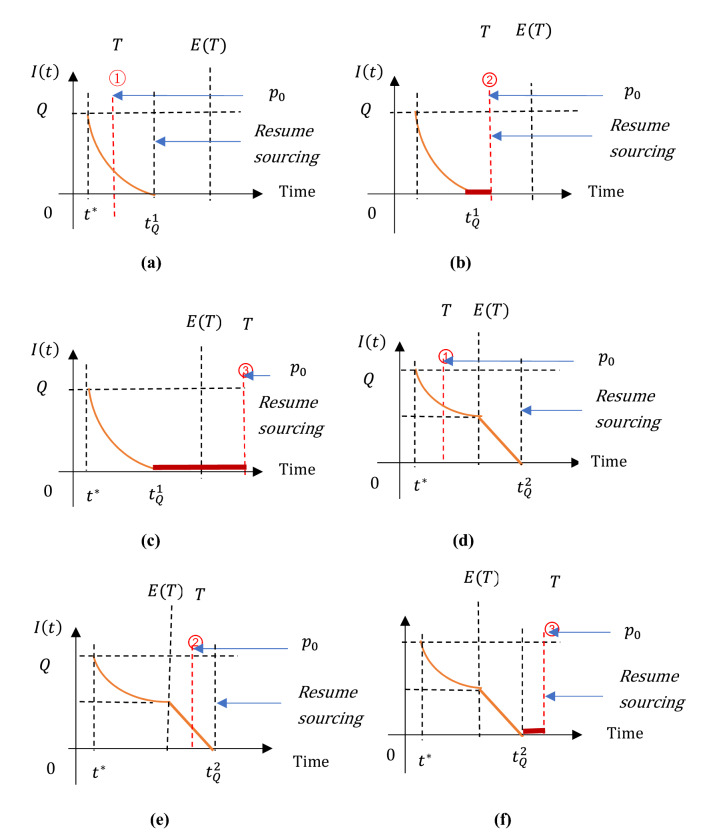
Manufacturer’s inventory $I\left(t\right)$ under contingent sourcing $Q({t}^{*})$ in Case 2

The inventory held by the manufacturer exhibits complicated patterns, depending on customers stockpiling behavior and the time and quantity of contingent sourcing. We depict the inventory dynamics in Figs. 2 and 3, respectively for Cases 1–2. The manufacturer’s profits gaining from contingent sourcing are described accordingly.

(i) In Case 1

Without stockpiling, demand maintains at $d\left(t\right)=1$. Therefore, as depicted in Fig. 2, the manufacturer’s inventory $I\left(t\right)$ drops at the constant rate “$1$” until it reaches zero at time ${t}^{*}+Q$. Then, the manufacturer purchase from the main supplier once she/he is available. On the other hand, the selling price stops increasing and returns to ${p}_{0}$ immediately at the end of supply disruption.

Compared with the decision of doing nothing but passively waiting for supply restoration, the manufacturer achieves an extra sale revenue ${R}_{1}$ in the period $[{t}^{*},\mathrm{min}\{T,{t}^{*}+Q\}]$ by selling products at an increased selling price $p\left(t\right)={p}_{0}+\theta t$. However, to resume production at the time ${t}^{*}$, two costs are incurred: the contingent sourcing cost ${S}_{1}$ generated from the mark-up price of contingent sources, and the inventory holding cost ${I}_{1}$.

Summing up, the manufacturer’s expected profit $E(\Delta {P}_{1})$ is derived, as stated in Table 3.

(ii) In Case 2

**Table 3:** Manufacturer’s profits under contingent sourcing

Case		The extra profits gained from contingent sourcing $Q({t}^{*})$	
1		${I}_{1}$	${c}_{\mathrm{H}}{Q}^{2}/2$
		${S}_{1}$	${c}_{\mathrm{s}}Q$
		${R}_{1}$	${R}_{1}={R}_{1}^{a}={\int }_{{t}^{*}}^{T}A\mathrm{d}t$ if ${t}^{*}\le T\le Q+{t}^{*}$; ${R}_{1}={R}_{1}^{b}={\int }_{{t}^{*}}^{Q+{t}^{*}}A\mathrm{d}t$ if $Q+{t}^{*}<T\le M$
		$E(\Delta {P}_{1})$	${\int }_{{t}^{*}}^{Q+{t}^{*}}{f}_{T}{R}_{1}^{a}\mathrm{d}T+{\int }_{Q+{t}^{*}}^{M}{f}_{T}{R}_{1}^{b}\mathrm{d}T-{I}_{1}-{S}_{1}$
2	Scenario 1	${I}_{2}$	$c_{{\text{H}}} \mathop \smallint \limits_{{t^{*} }}^{{t_{Q}^{1} }} \left( {Q - \mathop \smallint \limits_{{t^{*} }}^{t} d\left( t \right){\text{d}}t} \right){\text{d}}t$
		${S}_{2}$	${c}_{s}Q$
		${R}_{2}$	${R}_{2}={R}_{2}^{1a}$ if ${t}^{*}\le T\le {t}_{Q}^{1}$; ${R}_{2}={R}_{2}^{1b}$ if ${t}_{Q}^{1}<T\le M$
		$E(\Delta {P}_{2})$	${\int }_{{t}^{*}}^{{t}_{Q}^{1}}{f}_{T}{R}_{2}^{1a}\mathrm{d}T+{\int }_{{t}_{Q}^{1}}^{M}{f}_{T}{R}_{2}^{1b}dT-{I}_{2}-{S}_{2}$
	Scenario 2	${I}_{2}$	$c_{{\text{H}}} \left[ {\mathop \smallint \limits_{{t^{*} }}^{E\left( T \right)} \left( {Q - \mathop \smallint \limits_{{t^{*} }}^{t} d\left( t \right){\text{d}}t} \right){\text{d}}t + \frac{1}{2}\left( {t_{Q}^{2} - E\left( T \right)} \right)^{2} } \right]$
		${S}_{2}$	${c}_{s}Q$
		${R}_{2}$	${R}_{2}={R}_{2}^{2a}$ if ${t}^{*}\le T\le E(T)$; ${R}_{2}={R}_{2}^{2b}$ if $E\left(T\right)<T\le {t}_{Q}^{2};$ ${R}_{2}={R}_{2}^{2c}$ if ${t}_{Q}^{2}<T\le M$
		$E(\Delta {P}_{2})$	${\int }_{{t}^{*}}^{E\left(T\right)}{f}_{T}{R}_{2}^{2a}\mathrm{d}T+{\int }_{E\left(T\right)}^{{t}_{Q}^{2}}{f}_{T}{R}_{2}^{2b}\mathrm{d}T+{\int }_{{t}_{Q}^{2}}^{M}{f}_{T}{R}_{2}^{2c}\mathrm{d}T-{I}_{2}-{S}_{2}$

As afore-described in Fig. 1, due to the customers’ stockpiling behavior, demand maintains at $d\left(t\right)={d}_{1}(t)=2-\frac{2}{E(T)}t$ during the period $[t, E(T)]$ and $d\left(t\right)=1$ thereafter. Accordingly, the manufacturer’s inventory $I\left(t\right)$ falls into two scenarios, depending on the critical time point when inventory is entirely depleted.

- In **Scenario 1** where $Q\le {\int }_{{t}^{*}}^{E(T)}{d}_{1}(t)\mathrm{d}t$: the manufacture purchases a relatively small amount of contingent sources. As a result, the inventory drops at the demand rate ${d}_{1}(t)$ and reaches zero at the time point ${t}_{Q}^{1}$. Here, ${t}_{Q}^{1}$ is defined by the equation ${\int }_{{t}^{*}}^{{t}_{Q}^{1}}{d}_{1}(t)\mathrm{d}t=Q$, and ${t}_{Q}^{1}<E(T)$ (see Figs.3(a-c)).
- In **Scenario 2** where $Q>{\int }_{{t}^{*}}^{E(T)}{d}_{1}(t)\mathrm{d}t$: the manufacture purchases massive contingent sources, leading to a positive inventory of raw material left in the production system at the time $E(T)$. Then, inventory is consumed at the rate “$1$” until it reaches zero at the time ${t}_{Q}^{2}= E\left(T\right)+[Q-{\int }_{{t}^{*}}^{E\left(T\right)}{d}_{1}(t)\mathrm{d}t]$ (see Fig. 3d–f).

Similar to Case 1, based on the inventory dynamics stated above, the manufacturer’s expected profit $E(\Delta {P}_{2})$ that is composed of the extra sale revenue ${R}_{2}$ and the costs ${I}_{2}$ and ${S}_{2}$ of inventory holding and contingent sourcing, can be generated for Case 2. Table 3 summarizes $E(\Delta {P}_{2})$ arrived in both Scenarios 1 and 2.

Where, ${c}_{1}$ is the unit production cost and ${f}_{T}$ represents the probability density function of the random variable $T$. $A={p}_{0}+\theta t-{c}_{1}$, ${R}_{2}^{1a}={\int }_{{t}^{*}}^{T}d\left(t\right)A\mathrm{d}t$, ${R}_{2}^{1b}={\int }_{{t}^{*}}^{{t}_{Q}^{1}}d\left(t\right)A\mathrm{d}t$, ${R}_{2}^{2a}={\int }_{{t}^{*}}^{T}d\left(t\right)A\mathrm{d}t$, ${R}_{2}^{2b}={\int }_{{t}^{*}}^{E\left(T\right)}d\left(t\right)A\mathrm{d}t+{\int }_{E\left(T\right)}^{T}A\mathrm{d}t$, and ${R}_{2}^{2c}={\int }_{{t}^{*}}^{E\left(T\right)}d\left(t\right)A\mathrm{d}t+{\int }_{E\left(T\right)}^{{t}_{Q}^{2}}A\mathrm{d}t$.

### The optimal sourcing policy

Based on the calculation of $E(\Delta {P}_{1})$ and $E(\Delta {P}_{2})$ presented in Table 3, the model for determining the optimal contingent sourcing time ${t}^{*}$ and quantity $Q$, with the objective of maximizing the manufacturer’s profit, can be formulated as follows.

8 $$\left({t}^{*},Q\right)\in \mathrm{argmax}\left\{E\left(\Delta {P}_{1}\right), E\left(\Delta {P}_{2}\right)\right\}.$$

9 $$ {\text{Subject to }}t^{*} \ge 0\quad {\text{and}}\quad Q \ge 0. $$

where (9) guarantees the non-negativities of the decision variables. In particular, if ${t}^{*}=0$, a policy of instantaneous contingent sourcing is superior at the occurrence of the supply failure. Otherwise, it indicates that it is advisable to wait for some time before contingent sourcing.

Due to the complex expressions of $E(\Delta {P}_{1})$ and $E(\Delta {P}_{2})$, problem (8)–(9) cannot be analytically examined. Therefore, we next investigate the optimal contingent sourcing policy via numerical analysis.

To simplify the analysis, we follow a common assumption that the disruption length $T$ follows a uniform distribution on the interval $[0, 2E(T)]$ [37]. That is, the probability density function of the random variable $T$ is determined as ${f}_{T}=1/M$. Here, $M=2E(T)$. Note, the present assumption represents the following common case: the upper bound of the disruption length $T$ is acknowledged to the manufacturer. However, he/she has no access to more accurate information to conduct a more precise prediction on $T$.

Then, by establishing a basic set of parameter values as: ${p}_{0}=5,{c}_{1}=1, { c}_{\mathrm{s}}=1,{ c}_{\mathrm{h}}=0.5,{ c}_{\mathrm{H}}=1, \theta =1.5$, we solve the problem (8)–(9) under the giving setting. The optimal sourcing policy that indicates sourcing time ${t}^{*}$ and quantity $Q$ for disruption with a mean length $E(T)$ can be obtained accordingly. We let the relevant parameters vary and observe how the optimal joint decision $({t}^{*},Q)$ will change. Note that, we focus on the variation trends of relative factors and have run abundant analysis on other basic values of these factors. Our main findings generated from the given setting would not change.

**Fig. 4:**
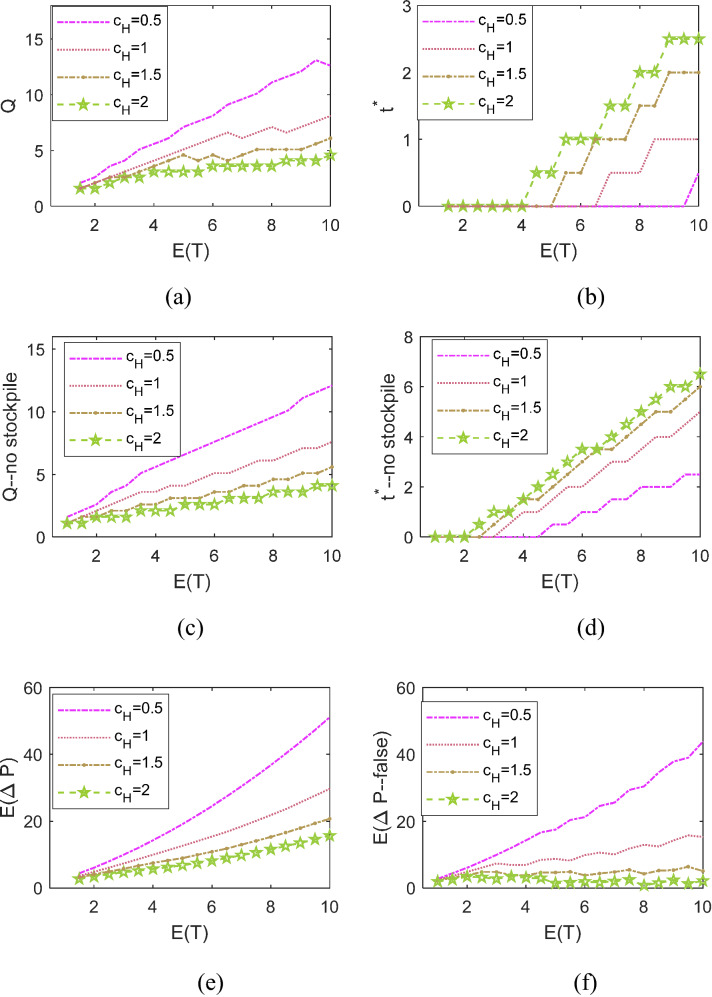
The optimal dynamic sourcing policies under different ${c}_{\mathrm{H}}$, with and without considering customers’ stockpiling behavior

Figure 4a, b show the optimal sourcing time ${t}^{*}$ and quantity $Q$ to cope with disruptions with different mean values of length, under different inventory holding costs ${c}_{\mathrm{H}}$. The *x* axis represents the mean value of disruption length. Several main findings are indicated.

First, Fig. 4a reveals that a larger $Q$ is suggested in coping with longer disruptions, or if the inventory holding cost ${c}_{\mathrm{H}}$ is less. In particular, if ${c}_{\mathrm{H}}$ becomes extremely small, the optimal sourcing quantity could exceed $E(T)$. In other words, facing significant advantages in sourcing cost, we suggest that the manufacturer procures massive contingent sources to cope with the uncertainty that the disruption length might exceed the mean value with a probability of 50%. By doing so, he/she could arrive at more profits once the situation occurs. Second, as shown in Fig. 4b, it is optimal to employ contingent sourcing immediately at the beginning (i.e., ${t}^{*}=0$) in mitigating short supply disruptions, and to wait for some time before rerouting for contingent replenishments (i.e., ${t}^{*}>0$) if the supply failure lasts long. We also observe that the advantage of such transient sourcing decision diminishes with ${c}_{\mathrm{H}}$. Thus, third, the sourcing time exhibits two trends as ${c}_{\mathrm{H}}$ increases: the instantaneous contingent sourcing becomes inadvisable for shorter disruptions; the manufacturer should wait for longer periods in the process of hedging against long disruptions.

To address further investigation on how the customers’ stockpiling behavior affects the manufacturer’s sourcing decision, we further examine the optimal decision $({t}^{*},Q)$ without taking into consideration the customers’ stockpiling behavior. That is, assuming that demand always remains at $d(t)=1$, a serial of false-optimal joint decisions is depicted in Fig. 4c, d. Comparing the results presented in Fig. 4a, b with the false-optimal joint decisions, we find that the manufacturer reroutes to the secondary sources later than the optimal time point if he/she neglects customers’ stockpiling. As a result, a significant loss in profit is incurred. Note, the *y* axes in Fig. 4e, f respectively stand for the manufacturer’s profits with and without considering customers’ stockpiling behavior.

**Fig. 5:**
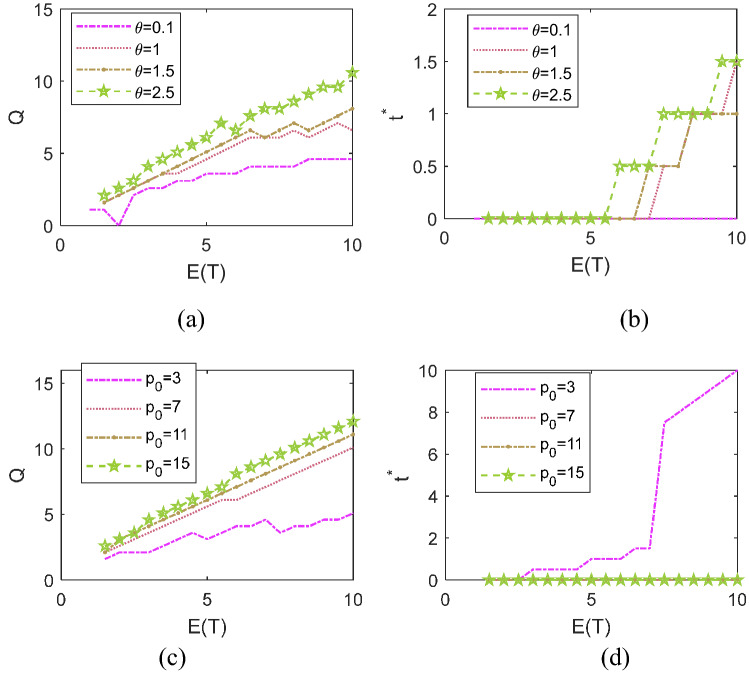
The optimal dynamic sourcing policies under different $\theta$ and ${p}_{0}$

Figure 5 depicts the insights on how to adjust optimal dynamic sourcing policies in accordance with the two aspects of the selling price: the pre-disruption selling price ${p}_{0}$ and the price increase rate $\theta$ during disruption. In general, growth in selling price, no matter before or after the presence of disruption (i.e., ${p}_{0}$ or $\theta$), leads to an increase in sourcing quantity. On the other hand, the variation trends of the optimal sourcing time with these two price factors reveal several different findings.

First, the decision of instantaneous sourcing is advantageous if $\theta$ is small. A higher $\theta$ leads to a larger sourcing time in mitigating long disruptions. Second, if ${p}_{0}$ is small, the optimal sourcing time is determined as ${t}^{*}\ge E(T$) for long disruptions. That is, under this circumstance, we suggest that the manufacturer allows lost sales before time $E(T$) and employ contingent sourcing to satisfy demands arriving thereafter if the disruption length actually exceeds the mean value. The non-intuitive reason is stated as follows. Due to customers’ stockpiling behavior, the demand rate initiates from the maximum value and drops over time during $(0, E(T$)). Therefore, when the selling price is not profitable enough, it could be better for the manufacture to contingently resume production after the demand rate returns to “1” after time $E(T$). On the contrary, once the pre-disruption selling price of the product exceeds a certain level, it is always preferable to implement instantaneous sourcing at the beginning of the supply failure, no matter whether or not the selling price will significantly grow, or how long the disruption will last.

## Conclusions

In this paper, we consider a maker-to-order system where a manufacturer halts production due to a supply disruption. There are competing products in the market that customers will turn into during stock-outs. Based on identifying customer stockpiling behavior, we investigate dynamic contingent sourcing strategies for the manufacturer.

By characterizing customer utility under possible selling price rises and expected disruption duration, we analytically illustrate that customer post-disruption stockpiling behavior falls into three patterns: non-stockpiling, gradual stockpiling, and instantaneous stockpiling. If the disruption lasts short and no significant price rise appears during disruption, non-stockpiling is suggested for customers. Conversely, if the disruption will last significantly short and the selling price will rise sharply, instantaneous stockpiling of the products required for the entire disruption is performed. In general occasions other than these two cases, stockpiling should be gradually adopted at a medium speed.

In view of the complicated cases regarding the dynamics of customer stockpiling behavior, the manufacturer decides to order from a second supply source. To this end, we establish the dynamic patterns of the demand and inventory with closed forms. A model for developing dynamic contingent sourcing strategies is then formulated, identifying the optimal joint decision of sourcing time and quantity to maximize the profit generated after the occurrence of disruption.

By conducting numerical analyses, we provide two managerial suggestions for practitioners. First, in hedging against short disruptions, we suggest that the manufacturer acquire replenishments from a secondary supplier instantaneously at the appearance of a supply failure in many circumstances. However, the advantage of instantaneous sourcing diminishes on the following two occasions: price grows significantly during disruption, or inventory holding cost is large. For long disruptions, we suggest that the manufacturer waiting some time before contingent sourcing. Second, the according sourcing quantity is suggested to be less for a higher inventory holding cost, a shorter disruption, or a low selling price. In addition to specific suggestions, we visually indicate how the two components of price, i.e., the pre-disruption price and the increased rate of price during disruption, play different roles in the optimal sourcing time. The profit loss that can be incurred from neglecting customer stockpiling behavior during the process of designing mitigation strategies, is presented as well.

The present study suggests several relative topics for future research. First, we consider a linear assumption when characterizing the dynamics of customer stockpiling behavior in this paper. Although the discussion extends the identification of customer stockpiling behavior from static descriptions to a dynamic pattern, it would be valuable to further extend this assumption to other possible patterns. Second, the random disruption is subjected to a uniform distribution here, capturing the uncertainty of disruption to a certain extent. To present more widely applicable sourcing strategies, another area worthy of exploration is to characterize disruptions via more types of general distributions.

## Figures and Tables

**Fig. 6:**
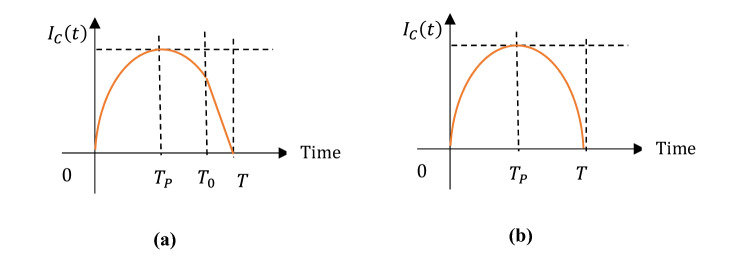
The corresponding inventory ${I}_{\mathrm{C}}\left(t\right)$ under $d\left(t\right)=b-at$

## Appendix: Calculation for Table 2

In accordance with Fig. 1, the inventory dynamics for Patterns 1 and 2 are presented in Fig. 6.

(i) In Pattern 1 where ${T}_{0}\le E(T)$:

Based on the dynamics of inventory and demand given in Figs. 1 and 6, we have

10 $$ I_{{\text{C}}} \left( t \right) = \left\{ {\begin{array}{*{20}l} {\mathop \smallint \limits_{0}^{t} \left( {b - a\tau - 1} \right){\text{d}}\tau = \left( {b - 1} \right)t - \frac{1}{2}at^{2} , } \hfill & { {\text{if}}\; 0 \le t \le T_{0} ;} \hfill \\ {\left( {b - 1} \right)T_{0} - \frac{1}{2}aT_{0}^{2} - \left( {t - T_{0} } \right) = bT_{0} - \frac{1}{2}aT_{0}^{2} - t,} \hfill & {{\text{if}}\; T_{0} < t \le E\left( T \right).} \hfill \\ \end{array} } \right. $$

Thus, the customers’ expected utility (2) can be further specified as:

11 $${U}_{1}={\int }_{0}^{{T}_{0}}\left({v}_{0}-{p}_{0}-\theta t\right)\left(b-at\right)\mathrm{d}t-{c}_{\mathrm{h}}{\int }_{0}^{{T}_{0}}\left[\left(b-1\right)t-\frac{1}{2}a{t}^{2}\right]\mathrm{d}t-{c}_{\mathrm{h}}{\int }_{{T}_{0}}^{E\left(T\right)}\left(b{T}_{0}-\frac{1}{2}a{{T}_{0}}^{2}-t\right)\mathrm{d}t.$$

(ii) In Pattern 2 where ${T}_{0}>E(T)$:

Similarly, the customers’ expected utility in Pattern 2 is derived as:

12 $${U}_{2}={\int }_{0}^{E(T)}\left({v}_{0}-{p}_{0}-\theta t\right)\left(b-at\right)\mathrm{d}t-{c}_{\mathrm{h}}{\int }_{0}^{E\left(T\right)}\left[\left(b-1\right)t-\frac{1}{2}a{t}^{2}\right]\mathrm{d}t.$$

As indicated in (11) and (12), both ${U}_{1}$ and ${U}_{2}$ are linear to the parameters $a$ and $b$. Thus, the maximization of ${U}_{1}$ and ${U}_{2}$ can only be achieved at the boundaries of the linear function $d\left(t\right)=b-at$, that is, Cases 1–3.

Next, we compare the customers’ expected utilities on these boundaries to determine the optimal decision of customers. By substituting the expressions of $d\left(t\right)$ (see Fig. 1) into (2), the customers’ expected utilities of Cases 1–3 are derived, denoted as ${U}_{1}$, ${U}_{2}$, and ${U}_{3}$, respectively.

13 $${U}_{1}={\int }_{0}^{E(T)}\left({v}_{0}-{p}_{0}-\theta t\right)\mathrm{d}t=\left({v}_{0}-{p}_{0}\right)E\left(T\right)-\frac{1}{2}\theta {E\left(T\right)}^{2}.$$

$${U}_{2}={\int }_{0}^{T}\left({v}_{0}-{p}_{0}-\theta t\right)\left(b-at\right)\mathrm{d}t-{c}_{h}{\int }_{0}^{T}\left[\left(b-1\right)t-\frac{1}{2}a{t}^{2}\right]\mathrm{d}t$$

14 $$=\left({v}_{0}-{p}_{0}\right)E\left(T\right)-\theta {E\left(T\right)}^{2}+\frac{2}{3}\theta {E\left(T\right)}^{3}-\frac{1}{6}{E\left(T\right)}^{2}{c}_{\mathrm{h}}.$$

15 $${U}_{3}=\left({v}_{0}-{p}_{0}\right)E\left(T\right)-\frac{1}{2}{c}_{\mathrm{h}}{E\left(T\right)}^{2}.$$

Note, $b=2$ and $a=2/E(T)$ are determined for $d\left(t\right)=b-at$ in Case 2.

Accordingly, the differences in customers’ utilities are determined. Furthermore, we see that:

16 $$ U_{2} - U_{3} = - \theta E\left( T \right)^{2} + \frac{2}{3}\theta E\left( T \right)^{3} + \frac{1}{3}c_{{\text{h}}} E\left( T \right)^{2} > 0. \leftrightarrow E\left( T \right) > \frac{3}{2} - \frac{{c_{{\text{h}}} }}{2\theta }. $$

17 $$ U_{2} - U_{1} = - \frac{1}{2}\theta TE\left( T \right)^{2} + \frac{2}{3}\theta E\left( T \right)^{3} - \frac{1}{6}E\left( T \right)^{2} c_{{\text{h}}} > 0. \leftrightarrow E\left( T \right) < \frac{3}{4} + \frac{{c_{{\text{h}}} }}{4\theta }. $$

18 $$ U_{3} - U_{1} = - \frac{1}{2}c_{{\text{h}}} E\left( T \right)^{2} + \frac{1}{2}\theta E\left( T \right)^{2} . \leftrightarrow \theta > c_{{\text{h}}} . $$

19 $$ \frac{3}{2} - \frac{{c_{{\text{h}}} }}{2\theta } > 1 > \frac{3}{4} + \frac{{c_{{\text{h}}} }}{4\theta }\quad {\text{if}}\;\theta > c_{{\text{h}}} ,\quad {\text{and}}\quad \frac{3}{2} - \frac{{c_{{\text{h}}} }}{2\theta } < 1 < \frac{3}{4} + \frac{{c_{{\text{h}}} }}{4\theta }\quad {\text{if}}\;\theta < c_{{\text{h}}} . $$

Summing up (16)–(19), Table 4 is achieved.

**Table 4:** The comparison of customers’ utilities in Cases 1–3

Scenarios of comparison			Optimal decision	Condition of optimal decision
${U}_{3}>{U}_{1}$: $\theta >{c}_{\mathrm{h}}$	${U}_{2}>{U}_{3}$	${U}_{2}>{U}_{1}$	Case 2 with ${U}_{2}$	$E(T)>\frac{3}{2}-\frac{{c}_{\mathrm{h}}}{2\theta }$
	${U}_{2}<{U}_{3}$	${U}_{2}<{U}_{1}$	Case 3 with ${U}_{3}$	$E(T)< \frac{3}{4}+\frac{{c}_{\mathrm{h}}}{4\theta }$
	${U}_{2}<{U}_{3}$	${U}_{2}>{U}_{1}$	Case 3 with ${U}_{3}$	$\frac{3}{4}+\frac{{c}_{\mathrm{h}}}{4\theta }<E(T)<\frac{3}{2}-\frac{{c}_{\mathrm{h}}}{2\theta }$
${U}_{3}<{U}_{1}$: $\theta <{c}_{\mathrm{h}}$	${U}_{2}>{U}_{3}$	${U}_{2}>{U}_{1}$	Case 2 with ${U}_{2}$	$E(T)> \frac{3}{4}+\frac{{c}_{\mathrm{h}}}{4\theta }$
	${U}_{2}<{U}_{3}$	${U}_{2}<{U}_{1}$	Case1 with ${U}_{1}$	$E(T)<\frac{3}{2}-\frac{{c}_{\mathrm{h}}}{2\theta }$
	${U}_{2}>{U}_{3}$	${U}_{2}<{U}_{1}$	Case1 with ${U}_{1}$	$\frac{3}{2}-\frac{{c}_{\mathrm{h}}}{2\theta }<E(T)< \frac{3}{4}+\frac{{c}_{\mathrm{h}}}{4\theta }$

Table 2 is directly deduced from Table 4.
